# Monthly Cost-Sharing Limits and Out-of-pocket Costs for Commercially Insured Patients in the US

**DOI:** 10.1001/jamanetworkopen.2022.33006

**Published:** 2022-09-15

**Authors:** Paul R. Shafer, Michal Horný, Stacie B. Dusetzina

**Affiliations:** 1 Department of Health Law, Policy, and Management, School of Public Health, Boston University, Boston, Massachusetts; 2 Department of Radiology and Imaging Sciences, School of Medicine, Department of Health Policy and Management, Rollins School of Public Health, Emory University, Atlanta, Georgia; 3 Department of Health Policy, Vanderbilt University School of Medicine, Nashville, Tennessee

## Abstract

This cross-sectional study assesses how much monthly cost-sharing limits, as opposed to annual limits, could reduce out-of-pocket costs for commercially insured patients in the US.

## Introduction

The Patient Protection and Affordable Care Act (ACA) aimed to improve affordability by requiring that all Marketplace and ACA-compliant health insurance plans include an annual maximum for in-network out-of-pocket costs. Despite this, difficulty affording care has remained a problem even for the insured, many of whom cannot afford their deductibles.^1,2,3^ Less than one-quarter of households with incomes between 150% and 400% of the federal poverty level can afford a typical family deductible of $12 000.^1^ High out-of-pocket costs can cause downstream adverse health effects, such as delayed or missed care and poor medication adherence.^3,4^ Policy makers have focused on addressing out-of-pocket costs rather than high prices for health care and prescription drugs. For example, President Biden proposed annual caps on retail drug costs in Medicare and monthly caps for insulin in his Build Back Better Framework, both of which were passed by Congress in the Inflation Reduction Act in August 2022. We extend this idea to in-network care received by the commercially insured. Our objective is to assess how much monthly cost-sharing limits, as opposed to annual limits, could reduce patients’ out-of-pocket costs.

## Methods

This cross-sectional study follows the STROBE reporting guidelines and was considered exempt by Boston University Medical Campus institutional review board. We used national commercial health insurance claims data from the 2019 IBM MarketScan Commercial Database to assess changes in patient cost-sharing and plan spending under hypothetical monthly limits.^5^ We restricted the sample to those younger than age 65 years, enrolled for the full calendar year, and not enrolled in a health maintenance organization or capitated plan. We recalculated patient and plan costs under a $250 and $500 monthly limit for in-network out-of-pocket costs across all claims (inpatient, outpatient, and pharmacy) using the observed values as our status quo. We then collapsed at the patient-month (highest monthly in-network out-of-pocket costs) and patient-year (in-network out-of-pocket costs, out-of-network out-of-pocket costs, and plan costs) levels to obtain the outcomes used. We also calculated the percentage of enrollees benefitting under each monthly limit and the share of total costs shifted back to plans as a proxy for how much premiums would have to increase, assuming health care use and prices remained constant. Finally, we stratified by enrollment in a low-deductible vs high-deductible health plan because we expect those with higher deductibles to benefit more. Data analysis was performed from June 2020 to July 2022 using SAS statistical software version 9.4 (SAS Institute) and Stata statistical software version 16.1 (StataCorp).

## Results

Instituting a $500 monthly limit for in-network out-of-pocket costs was estimated to affect nearly one-quarter (24.1%) of the commercially insured (Table 1). Median (IQR) annual in-network out-of-pocket costs decreased from $2024.66 ($1267.82-$3233.34) to $1102.61 ($720.73-$1628.05) (Table 1) among those with costs exceeding the cap, a 45.5% decrease. Enrollees in high-deductible health plans had greater savings (−49.9%) than those with lower deductibles (−44.1%). Median (IQR) highest monthly out-of-pocket costs for in-network care decreased from $1139.80 ($710.61-$2038.11) to $500.00 (by definition) (Table 1) among those affected, a 56.1% decrease. Avoided out-of-pocket costs would be shifted back to the plan, increasing annual mean plan costs per enrollee by 5.6% (Table 2), a proxy for how much premiums could be expected to increase. The reductions in out-of-pocket costs are even more pronounced with a $250 monthly limit for in-network care (Table 1 and Table 2).

**Table 1.** Projected Out-of-pocket and Plan Costs Under Hypothetical Monthly Cost-Sharing Limits, Among Affected Enrollees^a^

Scenario and population	Enrollees with reduced out-of-pocket costs, % (95% CI)	Annual out-of-pocket costs per enrollee for in-network care among those affected			Highest monthly out-of-pocket costs per enrollee for in-network care among those affected		
		Without monthly limit (status quo), median (IQR), $	With monthly limit, median (IQR), $	Change, $ (%)	Without monthly limit (status quo), median (IQR), $	With monthly limit, median (IQR), $	Change, $ (%)
$500 monthly limit on out-of-pocket costs for in-network care							
All enrollees	24.1 (24.0-24.1)	2024.66 (1267.82-3233.34)	1102.61 (720.73-1628.05)	−922.05 (−45.5)	1139.80 (710.61-2038.11)	500.00 (by definition)	−639.80 (−56.1)
Low-deductible health plan enrollees	21.7 (21.7-21.7)	1929.89 (1197.53-3056.51)	1079.60 (708.67-1596.61)	−850.29 (−44.1)	1067.44 (687.17-1900.88)		−567.44 (−53.2)
High-deductible health plan enrollees	29.5 (29.4-29.5)	2281.56 (1403.63-3500.00)	1143.87 (744.80-1678.92)	−1137.69 (−49.9)	1277.14 (760.25-2286.24)		−777.14 (−60.9)
$250 monthly limit on out-of-pocket costs for in-network care							
All enrollees	36.8 (36.8-36.9)	1405.00 (784.45-2550.65)	691.34 (439.00-1055.72)	−713.66 (−50.8)	702.69 (399.81-1478.99)	250.00 (by definition)	−452.69 (−64.4)
Low-deductible health plan enrollees	34.1 (34.1-34.1)	1325.73 (763.97-2352.52)	686.63 (436.43-1052.58)	−639.10 (−48.2)	660.12 (389.03-1348.68)		−410.12 (−62.1)
High-deductible health plan enrollees	43.0 (43.0-43.1)	1564.93 (829.32-2876.52)	700.14 (443.73-1061.20)	−864.79 (−55.3)	799.43 (422.79-1720.05)		−549.43 (−68.7)

^a^ Our identification of plan type relies on a MarketScan-provided measure from which high-deductible health plans with and without savings options (high-deductible health plan or consumer-driven health plan) are categorized as high deductible, and all other types (basic/major medical, comprehensive, exclusive provider organization, point-of-service plan, and preferred provider organization) are categorized as low deductible. Health maintenance organization and point-of-service plan with capitation were excluded. We weighted the data to be nationally representative of those with employer-sponsored coverage using IBM-provided weights derived from the American Community Survey (12 405 477 enrollees, representing a weighted population of 95 385 332 individuals).

**Table 2.** Projected Mean Annual Out-of-pocket and Plan Costs Under Hypothetical Monthly Cost-Sharing Limits, Among All Enrollees

Scenario	Annual cost per enrollee, mean (95% CI), $			Change in costs from status quo, $ (%)		
	In-network out-of-pocket costs	Out-of-network out-of-pocket costs	Plan costs	In-network out-of-pocket costs	Total out-of-pocket costs	Plan costs
Status quo	800.23 (799.43-801.02)	79.45 (79.05-79.86)	5126.55 (5111.47-5141.63)	NA	NA	NA
$500 monthly limit on out-of-pocket costs for in-network care	510.65 (510.26-511.03)	79.45 (79.05-79.86)	5416.13 (5400.86-5431.39)	−289.58 (−36.2)	−289.58 (−32.9)	289.58 (5.6)
$250 monthly limit on out-of-pocket costs for in-network care	394.20 (393.92-394.48)	79.45 (79.05-79.86)	5532.58 (5517.27-5547.880)	−406.03 (−50.7)	−406.03 (−46.2)	406.03 (7.9)

Abbreviation: NA, not applicable.

## Discussion

The findings of this cross-sectional study suggest that capping monthly out-of-pocket costs could meaningfully reduce the cost-sharing burden for many commercially insured patients in the US without regulating health care prices. Others exploring the concept of resetting deductibles have found potential value gains, particularly for lower-income patients.^6^ On the other hand, moral hazard might increase inefficient health care use as a result of lower cost-sharing. A limitation of this study is that it holds spending constant and does not account for potential behavioral responses by patients to reduced cost-sharing obligations, which could increase overall health care spending. However, the preponderance of evidence suggests that underconsumption of health care because of cost is a substantially more pressing problem.^4^ Offering plans with smaller deductibles that reset over shorter time periods could help ease affordability while creating another dimension of patient choice.^2^ The resulting premium increase would be subsidized by employers or the government for the majority of US individuals, who are not responsible their full premiums, thereby making the large upfront cost for using health care more manageable.
